# Pneumatosis Intestinalis Following Surgical Gastrostomy Tube Placement in a Patient With Glottic Squamous Cell Carcinoma: A Case Report

**DOI:** 10.7759/cureus.60918

**Published:** 2024-05-23

**Authors:** Eli Zolotov, Caden Quintanilla, Noreen Ahmed, Anat Sigal, Zahf Shaikh, Davood K Hosseini, Robert Lee, Karan Omidvari, Nilesh Shukla

**Affiliations:** 1 Internal Medicine, Hackensack University Medical Center, Hackensack, USA; 2 Pediatrics, Hackensack University Medical Center, Hackensack, USA; 3 Gastroenterology, Hackensack University Medical Center, Hackensack, USA; 4 Critical Care, Hackensack University Medical Center, Hackensack, USA

**Keywords:** surgical gastrostomy placement complications, surgical gastrostomy placement, surgical gastrostomy, glottic squamous cell carcinoma, percutaneous endoscopic gastrostomy (peg), pneumatosis intestinalis

## Abstract

Pneumatosis intestinalis (PI) is a rare medical and post-surgical sequela of multiple different etiologies which can be either benign or life-threatening. Various mechanisms have been proposed to explain the occurrence of PI; however, the pathophysiology is dependent on the suspected cause. The condition is largely categorized into two broad groups: idiopathic PI, which remains relatively uncommon, and secondary PI. The latter often surfaces as a result of a wide array of both gastrointestinal and non-gastrointestinal illnesses. These encompass vascular compromise, bowel mucosal disruption, gastrointestinal dysmotility, as well as infectious and immunological etiologies. Management ranges from conservative medical strategies to emergent surgical intervention.

We present the first case to our knowledge of spontaneous PI developing within five days of a surgical gastrostomy tube (SGT) placement in a 79-year-old female with glottic squamous cell carcinoma which unfortunately proved fatal. The purpose of this case report is to highlight a rare fatal complication of a common surgical procedure and the necessity of initiating interdisciplinary management quickly to determine the best treatment course.

## Introduction

Pneumatosis intestinalis (PI) is a rare medical and post-surgical sequela of multiple different etiologies characterized by the presence of gas within the bowel walls. PI is predominantly benign, as observed in cases of connective tissue disorders including scleroderma and inflammatory bowel disease, as well as medication-induced cases involving corticosteroids. However, except for necrotizing enterocolitis, which is common in children, PI rarely poses a life-threatening risk [1]. The pathogenesis of PI is poorly understood and is likely multifactorial, with theories revolving around vascular compromise, bowel mucosal disruption, gastrointestinal dysmotility, as well as infectious and immunological etiologies [2-4]. PI's incidence and prevalence are hard to ascertain given its asymptomatic nature in most cases; however, it has previously been estimated at three per 10,000 individuals among the general population [5]. There are no definitive management guidelines for PI, which range from conservative medical management to urgent surgical intervention; various algorithms have been generated to aid the surgeon in this matter [6]. This report presents a fatal case of PI in a 79-year-old woman, identified after surgical gastrostomy tube (SGT) placement. This rare presentation of PI offers the potential connection between SGT placement and PI.

This case report was presented at the American Thoracic Society (ATS) Conference in May 2024.

## Case presentation

A 79-year-old woman with a past medical history of chronic obstructive pulmonary disease (COPD) and glottic squamous cell carcinoma (SCC) was referred to our medical center for oncologic evaluation and treatment. Following oncologic evaluation, the patient was determined not to be a candidate for chemotherapy due to her advanced glottic SCC and the severity of her comorbid conditions. The SCC prevented her from oral nutritional access by causing significant esophageal obstruction due to the glottic mass. This obstruction led to severe dysphagia as well as a high risk of aspiration. Multiple formal swallow evaluations confirmed that any attempt at oral intake could result in food or liquid aspiration, posing a severe risk of aspiration pneumonia and other respiratory complications. As a result of these evaluations, the patient was placed on nil per os (NPO) status. Consequently, she was not an appropriate candidate for nasogastric tube (NGT) placement or percutaneous endoscopic gastrostomy (PEG) placement. Given the complexity of providing nutrition to her, total parenteral nutrition was initiated on the eighth day of hospitalization, followed by SGT placement on the 13th day of hospitalization.

SGT placement was performed according to established protocols and best practices. The patient had a tracheostomy placed a few weeks prior to the procedure. Under general anesthesia, the patient was ventilated through the tracheostomy. The patient was positioned supine, and a short upper midline incision was made to identify the stomach. A site on the anterior wall near the greater curvature was chosen. Two silk purse-string sutures were placed on the stomach's anterior surface. A left upper quadrant skin incision was made, through which an 18-French gastrostomy tube was introduced. A gastrotomy was created within the purse-string sutures, and the gastrostomy tube was inserted into the stomach and secured. The stomach was anchored to the anterior abdominal wall at four points with silk sutures. The fascia was closed with polydioxanone sutures, and the skin was closed with staples. After the procedure, the patient was transitioned back to a tracheostomy collar. The patient was transferred from the operating room in stable condition, with no immediate postoperative complications.

**Figure 1:**
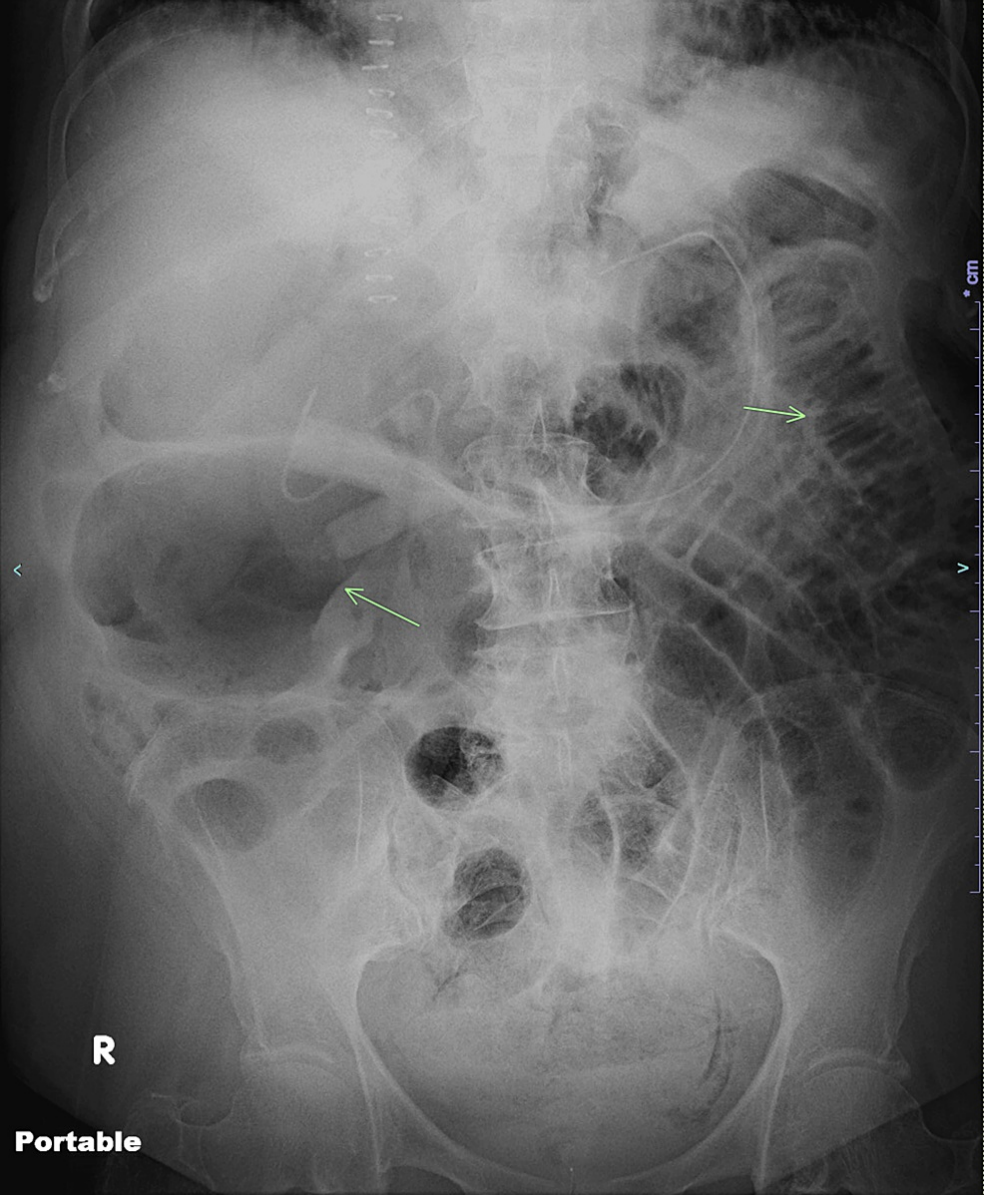
Abdominal radiograph. Anterior abdominal radiograph reveals clusters of mildly dilated small bowel loops in the left hemiabdomen. The right abdomen displays a prominently dilated, gas-filled bowel loop, which is likely corresponding to the ascending colon

**Figure 2:**
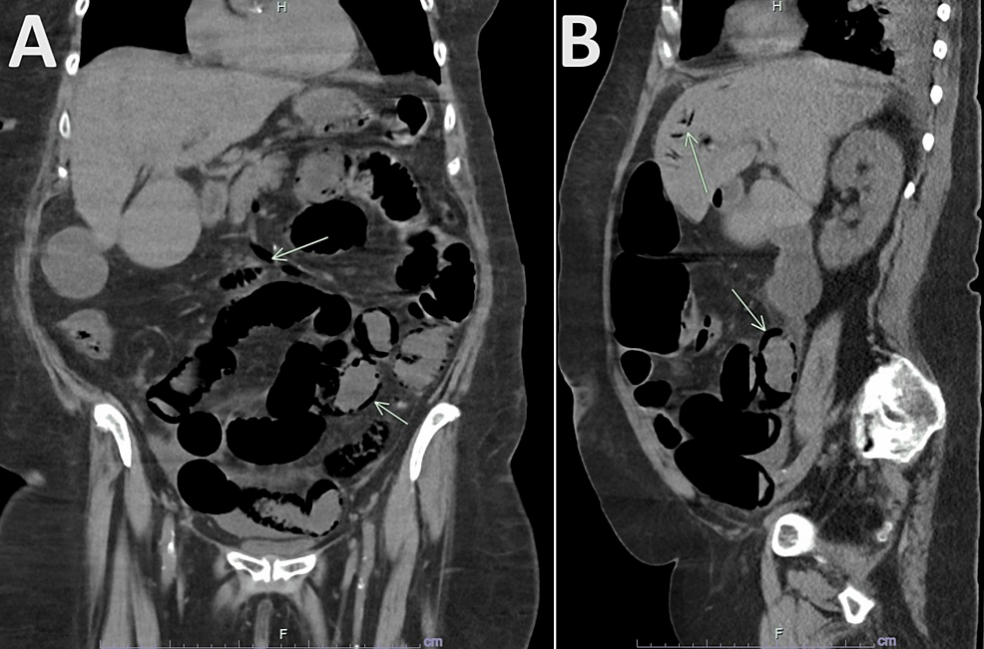
CT scan of the abdomen and pelvis without contrast. Sagittal (A) and coronal (B) sections show diffuse pneumatosis within the stomach, jejunum, and ileum with associated portal venous and superior mesenteric venous air

On postoperative day (POD) 1, the patient had stable vital signs with a heart rate of 84 beats per minute and a blood pressure of 154/66 mmHg. Her abdomen was soft, not distended, and appropriately tender in the incision area. However, the patient developed a fever of 101.1°F, and bilateral pneumonia was suspected. Consequently, broad-spectrum antibiotics, including piperacillin-tazobactam 4.5 g three times daily and vancomycin 1 g twice daily, were initiated. Sputum cultures collected on POD 3 identified *Proteus vulgaris*, with all other cultures testing negative. Consequently, two days later, the antimicrobial therapy was narrowed to ampicillin-sulbactam, administered at a dosage of 3 g three times daily. On POD 5, she developed acute-onset severe abdominal pain, during which laboratory tests were conducted (Table 1). Physical examination revealed a distended abdomen with bowel sounds in all four quadrants, tympanic resonance throughout, and diffuse tenderness. Abdominal X-ray was significant for dilated loops of the small bowel (Figure 1). Subsequent CT of the abdomen and pelvis without contrast indicated diffuse PI within the jejunal and ileal loops with associated portal venous and superior mesenteric venous air (Figure 2). Following the CT scan at 9 AM, a series of lactic acid tests were conducted, with levels recorded at 2.3 mmol/L at 11 AM, 2.2 mmol/L at 3 PM, and 2.1 mmol/L at 7 PM. Given the CT findings and the patient's advanced end-stage glottic SCC, a goals-of-care discussion was held with the patient and her family. This resulted in a change of her code status to "do not intubate" (DNI) and "do not resuscitate" (DNR). In the setting of changing the code status and transferring the patient to inpatient hospice care, it was decided to avoid further scanning with contrast-enhanced CT to exclude bowel ischemia. Subsequently, she passed away the following day.

**Table 1:** Laboratory data on postoperative day 5

Variable	Reference range	Postoperative day 5
Hematology		
Hemoglobin (g/dL)	13.0-17.0	10.0
Hematocrit (%)	36-46	30.6
White cell count (per μL)	4,000-11,000	22,000
Neutrophils (%)	40-75	93.3
Lymphocytes (%)	13-43	2.3
Monocytes (%)	0-13	4.0
Eosinophils (%)	0-5	0.1
Platelet count (per μL)	135,000-430,000	435,000
Chemistry		
Sodium (mmol/L)	136-145	140
Potassium (mmol/L)	3.5-5.1	3.9
Chloride (mmol/L)	98-107	107
Carbon dioxide (mmol/L)	22-29	22
Creatinine (mg/dL)	0.3-1.5	0.89
Urea nitrogen (mg/dL)	8-26	15
Aspartate aminotransferase (U/L)	5-34	32
Alanine aminotransferase (U/L)	0-55	14
Total bilirubin (mg/dL)	0.2-1.2	1.2
Glucose (mg/dL)	82-115	138
Lactate (mmol/L)	0.5-2.0	2.1

## Discussion

This case report highlights a correlation between SGT placement and PI. We suggest several theories that may explain this observation. Firstly, it may be due to bowel mucosal disruption [7,8]. The SGT placement may cause a degree of mechanical trauma to the bowel wall, which could potentially create a pathway for bacteria and gasses in the gastrointestinal tract to infiltrate into the bowel wall and cause PI. Secondly, it may be caused by vascular compromise [9,10]. Surgical manipulation and anesthesia during SGT placement could potentially disrupt the blood supply to the gastrointestinal tract, leading to areas of ischemia especially in watershed areas. Ischemic bowel walls are more susceptible to penetration and proliferation of bacteria and gasses from the gut, which may contribute to the development of PI. Thirdly, feeding after SGT placement could increase the demand for blood supply, especially in a setting of poor intestinal perfusion [11]. This can result in ischemic bowel and PI particularly after prolonged total parenteral nutrition use. 

A comparable case involved a 19-year-old patient with cerebral palsy who developed severe abdominal pain as a manifestation of PI 10 days after gastrostomy [12]. This case was elucidated by Perez Rivera et al., who hypothesized an intriguing pathophysiology. They suggested that severe malnutrition could disrupt carbohydrate digestion, thereby stimulating intestinal bacterial fermentation. This process could subsequently lead to an excessive gas build-up, potentially damaging the intestinal mucosal wall. The case was able to be managed conservatively with NGT placement, intravenous fluids, and holding his enteral nutrition with the patient improving after 24 hours. Another case, involving a 77-year-old female patient, presented PI two months post-PEG placement conducted via an endoscopic procedure [13]. However, the causative link between the PEG procedure and the subsequent onset of PI remains somewhat unclear in this instance. There is only one other documentation of pneumatosis after gastrostomy PEG placement noted in a retrospective analysis of 230 patients who underwent SGT placement [14]. In contrast to these reports, our case presents a more straightforward association, exhibiting PI as early as POD 5. This early manifestation diminishes the likelihood of attributing PI to other etiologies, such as anatomical alterations or gut microbiome changes.

However, other pathogenesis cannot be fully excluded. In the context of our patient, the PI's pathogenesis may involve a combination of the proposed mechanical, bacterial, and biochemical mechanisms. The mechanical theory might be applicable as the patient had COPD, which could lead to alveolar rupture and tracking of air along the mesenteric root to the bowel wall [15]. Also, the patient was on total parenteral nutrition, and the absence of regular enteral feeding might have altered the gut microbiota, potentially leading to excessive hydrogen gas production.

Bowel ischemia is also a potential contributor to the patient's PI. After identifying PI on the CT scan, a series of lactate measurements showed a downtrend in lactate levels, leading to a low suspicion of bowel ischemia. However, there are descriptions in the literature of patients with bowel ischemia who have normal lactate levels [16,17]. Therefore, even though contrast-enhanced CT was not performed due to the change in the patient's code status and transition to hospice care, bowel ischemia could still potentially develop and lead to PI. This is particularly relevant given the patient's advanced age, multiple comorbidities, and hypercoagulable state, which is caused by her postoperative status and underlying malignancy. These factors could contribute to superior mesenteric artery thrombosis, which, in turn, could lead to bowel ischemia and the formation of PI.

## Conclusions

The phenomenon of PI remains a rare medical phenomenon whose clinical implications are largely influenced by the specific clinical context and suspected underlying causes. This case report significantly contributes to the existing literature by establishing a more concrete link between surgical gastrostomy procedures and the development of PI as a postoperative complication. Furthermore, it underscores the critical nature of this condition, which, though infrequently, can lead to fatal outcomes. It is imperative that such cases receive prompt and effective interdisciplinary management to ensure that necessary surgical interventions are not delayed, thereby preventing potentially grave consequences.

## References

[1] Ho LM, Paulson EK, Thompson WM (2007). Pneumatosis intestinalis in the adult: benign to life-threatening causes. AJR Am J Roentgenol.

[2] Pear BL (1998). Pneumatosis intestinalis: a review. Radiology.

[3] Yale CE, Balish E, Wu JP (1974). The bacterial etiology of pneumatosis cystoides intestinalis. Arch Surg.

[4] van der Linden W, Marsell R (1979). Pneumatosis cystoides coli associated with high H2 excretion. Treatment with an elemental diet. Scand J Gastroenterol.

[5] Sato T, Ohbe H, Fujita M, Kushimoto S (2020). Clinical characteristics and prediction of the asymptomatic phenotype of pneumatosis intestinalis in critically ill patients: a retrospective observational study. Acute Med Surg.

[6] Khalil PN, Huber-Wagner S, Ladurner R (2009). Natural history, clinical pattern, and surgical considerations of pneumatosis intestinalis. Eur J Med Res.

[7] Koutouzis T, Lee J (2000). Blunt abdominal trauma resulting in pneumatosis intestinalis in an infant. Ann Emerg Med.

[8] Galea J, Burnand KM, Dawson FL, Sinha CK, Rex D, Okoye BO (2017). Pneumoperitoneum in the setting of pneumatosis intestinalis in children: is surgery always indicated?. Eur J Pediatr Surg.

[9] De Brauwer J, Masereel B, Visser R, Geyskens P (2006). Pneumatosis intestinalis caused by ischaemic bowel: report of three cases. Acta Chir Belg.

[10] Tahara S, Sakai Y, Katsuno H, Urano M, Kuroda M, Tsukamoto T (2019). Pneumatosis intestinalis and hepatic portal venous gas associated with gas-forming bacterial translocation due to postoperative paralytic ileus: a case report. Medicine (Baltimore).

[11] Fukunaga N, Yoshida S, Shimoji A (2022). Pneumatosis intestinalis and hepatic portal venous gas caused by enteral feeding after a heart valve surgery. J Cardiol Cases.

[12] Perez Rivera CJ, Ramirez NA, Gonzalez-Orozco A, Caicedo I, Cabrera P (2019). Pneumoperitoneum, pneumatosis intestinalis and portal venous gas: rare gastrostomy complications case report. Int J Surg Case Rep.

[13] Barbon Remis E, Garcia Pravia MP, Del Campo Ugidos RM, Garcia Álvarez C, Fernández Fernández MC (2017). Pneumomediastinum and pneumoperitoneum secondary to cystic intestinal pneumatosis after percutaneous endoscopic gastrostomy placement. Cir Esp.

[14] Vade A, Jafri SZ, Agha FP, Vidyasagar MS, Coran AG (1983). Radiologic evaluation of gastrostomy complications. AJR Am J Roentgenol.

[15] Iida A, Naito H, Tsukahara K (2017). Pneumatosis cystoides intestinalis presenting as pneumoperitoneum in a patient with chronic obstructive pulmonary disease: a case report. J Med Case Rep.

[16] Taylor J, Mandzhieva B, Shobar R (2020). Diagnosis of acute mesenteric ischemia in a patient with end-stage renal disease with normal serum lactate. Cureus.

[17] Demir IE, Ceyhan GO, Friess H (2012). Beyond lactate: is there a role for serum lactate measurement in diagnosing acute mesenteric ischemia?. Dig Surg.

